# Robotic guidance for percutaneous placement of triangular osteosynthesis in vertically unstable sacrum fractures: a single-center retrospective study

**DOI:** 10.1186/s13018-022-03489-4

**Published:** 2023-01-04

**Authors:** Zhao-Jie Liu, Ya Gu, Jian Jia

**Affiliations:** Department of Orthopaedics, Tianjin Hospital, 406 Jiefangnan Road, Tianjin, 300211 China

**Keywords:** Iliosacral screw, Robotic surgery, Sacral fracture, Minimally-invasive surgery, Fracture fixation, Internal

## Abstract

**Background:**

To evaluate the effectiveness and safety of robot-aided percutaneous triangular osteosynthesis combined with close reduction for vertically unstable sacrum fractures (VUSFs).

**Methods:**

The data on 21 patients of the VUSF were retrospectively analyzed from November 2016 to January 2021. According to Denis classification, there were 3 cases in zone I, 11 cases in zone II, and 7 case in zone III. The main perioperative indicators were recorded. The maximal angulation and displacement deviations of the screws were analyzed by comparing the planned trajectory with the actual position. Postoperative X-ray radiographs and CT scans were obtained for evaluating the reduction quality. Functional outcome was scored with Majeed criterion.

**Results:**

Fourteen patients of the unilateral VUSF and 7 patients of the bilateral VUSF underwent unilateral and bilateral triangular osteosynthesis with robotic assistance, respectively. No intraoperative neurovascular injuries and postoperative infection occurred. All patients were followed up for at least 12 months. The average operation time of posterior pelvic ring was 111.4 min, with the mean intraoperative bleeding of 110.5 ml. A total of 58 pedicle and iliosacral screws were implanted with robotic assistance. Of those, 52 screws were in the cancellous bone except 4 pedicle and 2 iliosacral screws cutting the cortical bone. The angulation and displacement deviations of the screws were 4.2° ± 2.5° and 1.7 ± 0.9 mm, respectively. The average displacement of the sacral fracture was reduced from 19.7 mm preoperatively to 3.1 mm postoperatively. According to Matta’s criterion, the reduction quality was graded as “excellent” in 13 patients and “good” in 8. All sacral fractures healed within 6 months except one fracture with nonunion. The mean Majeed score at the last follow-up was 89.6.

**Conclusions:**

Robot-aided triangular osteosynthesis combined with close reduction provide a safe and reliable option for percutaneous treatment of the fresh VUSF, with a high accuracy of iliosacral and pedicle screw implantation except insertion of iliac screws. Meanwhile, the technique may help to reduce incision-related complications.

## Background

Vertically unstable sacrum fractures (VUSFs) are commonly caused by high-energy violence, especially falls from height, and the majority of patients have concomitant injuries. As a severer pattern involving bilateral sacral foramen and canal, the multi-planar sacrum fracture can even result in spinopelvic instability and dissociation. The treatment aims to reconstruct the sacral anatomy and achieve sufficient stability in posterior pelvic ring for early mobilization.

Triangle osteosynthesis, containing the devices of a vertical vertebropelvic distraction osteosynthesis plus a horizontal fixation of the sacrum fracture with either an iliosacral screw or a trans-sacral plate, was first described for the treatment of VUSFs by Schildhauer et al. [1, 2]. With regards to fixation of sacral fractures with rotational and vertical instabilities, biomechanical studies have demonstrated that triangular osteosynthesis can provide better stiffness than single horizontal fixation, such as iliosacral screw and tension band plate fixation, hence enabling early weight-bearing [2, 3]. In addition to favorable biomechanical data, positive radiographic results of triangular osteosynthesis have also been demonstrated in a series of cases with vertically unstable sacrum fractures, with hardware loosening and failure rates as low as 5–9% [1, 4]. However, incision-related complications, including wound breakdown and infection are still concerned with conventional open reduction maneuvers [5]. Wound healing problems, particularly in patients with traumatic spinopelvic dissociation undertaken lumbopelvic fixation, were revealed to be troublesome, with the rate ranging from 14 to 26% [6, 7].

Although minimally invasive surgery has been increasingly developed in the treatment of posterior pelvic ring injuries, there is still a high amount of described hardware misposition via a fluoroscopy-guided free hand method, with the incidence of 10 to 15% [8, 9], resulting in vascular injuries and neurological sequalae, which meets the demand for a more reliable and precise technique of screw implantation. Technically, it is feasible to implant pedicle screws and iliosacral screws percutaneously with robotic assistance. In addition, precise screw implantation for both spinal and pelvic fixation can result in less invasion, thus reducing operation time and surgical bleeding, with the existing studies demonstrating that robot-aided orthopedic surgeries have significantly lower intraoperative fluoroscopy frequency than that with free hand procedure [10, 11]. However, there is a lack of literature investigating the application of triangle osteosynthesis under robotic guidance in patients with VUSFs.

The novel orthopedic robot, called as TiRobot (TINAVI Medical Technologies, China), has been produced and applied in China, representing the latest robot-based navigation system for orthopedic surgeries. This system can simulate the trajectory, the length and the direction of the implanted screw, with the positioning accuracy less than 1 mm. Until recently, robot-aided percutaneous triangular osteosynthesis has been performed in a number of patients with unilateral or bilateral VUSFs, and satisfactory results have been obtained after follow-ups in our institution. This study aims to evaluate the clinical efficacy and safety by analyzing the data of these cases.

## Methods

### Patients

Inclusion criteria were skeletally mature patients with displaced VUSFs revealed on radiological examinations, undertaking ipsilateral or bilateral percutaneous triangular osteosynthesis under robotic guidance within 3 weeks after injury, and patients with continuous follow-up period more than 12 months. Exclusion criteria were patients of the neurological damage treated with the sacral nerve or the sacral canal decompression, and with sacral dysmorphism in the vestibula that was not allowed to accommodate a *φ* 6.5 mm cannulated screw. According to the inclusion and exclusion criteria, retrospective data of 21 consecutive patients were collected in this study from November 2016 to January 2021. All procedures performed in this study were in accordance with the medical ethical standards of the institutional research committee.

After admission, appropriate radiographs were obtained immediately. Further image examinations, including an X-ray trauma series of pelvis (anteroposterior, inlet, and outlet views) and three-dimensional computed tomography (CT) reconstruction, were obtained routinely once patients were hemodynamically stable after fluid or blood infusion. According to the pelvic fracture, Tile classification for the stability of pelvic ring injuries, Denis classification for positioning of sacral fractures and Roy-Camille classification for the morphology of sacral fractures were noted in all patients. Depending on the type of posterior pelvic ring injuries, the ipsilateral distal femur traction was performed in patients of the unilateral VUSF, and patients of the bilateral VUSF underwent bilateral skeletal tractions. Once the conditions were medically stable, all VUSF patients underwent percutaneous triangular osteosynthesis with robotic assistance, and the surgical procedures were carried out by the same experienced surgeons.

### Surgical equipment

The 3rd generation TiRobot system, including a main console, an optical tracking system and a six-joint arm, was produced by TINAVI Medical Co., Ltd. in China. The intraoperative C-arm machine was produced by Siemens, Germany. All the implants, containing pedicle screws, iliac screws and cannulated screws, were made in China (Kanghui).

### Operative techniques

**Fig. 1:**
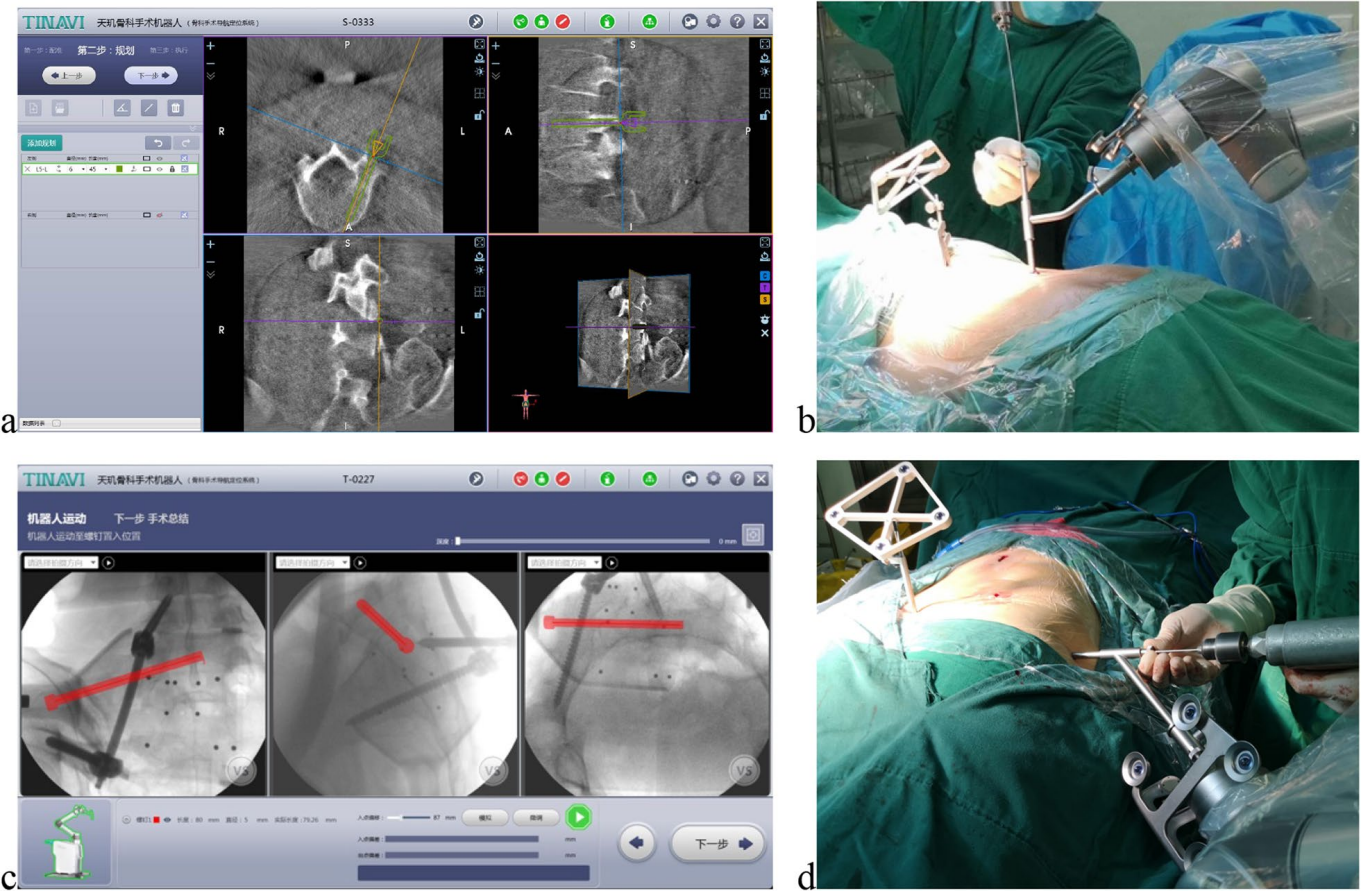
Intraoperative manipulation. **a** Planning of a L5 pedicle screw. **b** A guidewire drilling according to the planned pedicle trajectory under robotic assistance. **c** Planning of a S1 iliosacral screw. **d** A guidewire drilling according to the planned iliosacral trajectory under robotic assistance

1. Surgery is performed after general anesthesia, with the patient in a prone position on a radiolucent operation table. Then we covered the robotic arm with a sterile protective sleeve, installed a robot tracker with a calibrator at the end of the arm, and moved the arm to the disinfected surgical field.
2. A spinal tracker was clamped to the L3 spinous process through a 2-cm incision. After transmitting the intraoperative fluoroscopic data of the L5 vertebra to the main console, the trajectory and the dimension of the L5 pedicle screw on the affected side were planned by the operator (Fig. 1a). Prior to moving the robotic arm to the surgical area, the robot tracker was detached and replaced with a guider. Once the manipulation was confirmed, the spatial positioning orders for the robotic arm would be given. And then the guider moved automatically and located at the screw entry-point, keeping the planned angulation and direction. Next, the tip of a protected sleeve was inserted along the guider to contact the bone through a 1-cm incision, and a guidewire was then drilled into the L5 pedicle (Fig. 1b). In the process of drilling, the positioning accuracy displayed on the screen should be kept below 1-mm according to the prompt sent by the main console. After verification with fluoroscopy, a poly-axial cannulated pedicle screw was inserted, normally with the diameter of 60- to 65-mm and the length of 45- to 50-mm.
3. The ipsilateral posterior superior iliac spine (PSIS) was exposed via an oblique 3-cm incision. After establishment of the working corridor from PSIS to anterior inferior iliac spine (AIIS), a poly-axial iliac screw with the diameter of 6-mm and the length of 80- to 100-mm was implanted. A small osseous depression needs to be formed for iliac screw head accommodation, avoiding skin irritation. Then, a contoured rod with an appropriate length was inserted percutaneously to connect the pedicle screw and the iliac screw. Next, vertical migration of the hemipelvis was corrected by longitudinal traction combined with reduction tools such as distraction clamps and ball spike pushers, while a Schanz pin used as a joystick necessitated in rotational deformity. Once reduction was completed and verified with fluoroscopy, all connecters were locked.
4. In the process of planning the iliosacral screw, another tracker for limb positioning was fixed percutaneously at the contralateral PSIS. Intraoperative fluoroscopy, including pelvic inlet, outlet, and lateral views were obtained and transmitted to the main console. After drilling a guidewire into the S1 corridor according to the planned trajectory, a cannulated screw with the diameter of 7.3-mm was then inserted (Fig. 1c, d). It is essential to loosen and retighten the main connectors for relieving the longitudinal distracted pressure acting on the ipsilateral side of L5/S1 disc.
5. In the cases of the bilateral VUSF, pedicle and iliosacral screws on both sides can be planned simultaneously under TiRobot guidance.

### Postoperative management

Antibiotics weerere routinely administered in 48 h to prevent wound infection and low molecular weight heparin was subcutaneously injected for the prophylaxis of deep venous thrombosis. Appropriate mobilizations, such as sitting and active joint exercise in bed were encouraged at the following day after surgery. Postoperative radiographs and CT scans in pelvis were obtained to assess the reduction quality and screw position. Except for patients with lower extremity fractures, weight-bearing assisted by walking aids was allowed 2–4 weeks after surgery, and full weight-bearing exercise was commonly permitted if callus formation or fuzzy fracture lines were revealed on the X-ray plain films. Individualized rehabilitation plans were determined based on the treatment of concomitant injuries. Patients were followed up regularly once a month in the first half of the year and every 3 months thereafter.

According to the maximal displacement of fractures on three postoperative views, reduction quality was graded by Matta’s criterion with excellent (< 5 mm), good (5–10 mm), fair (11–20 mm), and poor (> 20 mm)[16]. At the final follow-up, functional results were evaluated using Majeed scoring system that was described as excellent (≥ 85), good (70–84), fair (55–69) and poor (< 55) [17].

### Statistical analysis

SPSS 20.0 software (Inc., Chicago, IL, USA)) was used for statistical analysis. Numerical data such as perioperative indicators were presented as mean ± standard deviation (SD).

## Results

Among the patients, 15 were caused by falling, 4 were involved by traffic accidents, and 2 suffered with crushing injuries (Table 1). Fourteen patients of the unilateral VUSF and 7 patients of the bilateral VUSF were categorized based on the CT images. Among bilateral VUSFs (i.e., traumatic spinopelvic dissociation), 6 sacral fractures were “U-shaped” and one was “H-shaped” according to the fracture morphology. Fractures or disruptions in L5/S1 facet joint were detected in 17 patients. Based on Tile classification [12], the study contained 12 patients of C1, 2 of C2, and 7 of C3. According to Denis classification [13], there were 3 cases in zone I, 11 in zone II, and 7 in zone III. Of those patients with sacral zone III involvement, one had Roy-Camille type I fracture, 3 had type II, 2 had type III and 1 had type IV [14]. Of all the patients, anterior pelvic ring fractures were associated in 20 patients, including rami fractures in 15 patients, the single symphyseal separation in 4 patients, and a combination of rami fractures and the symphyseal separation in one patient. Seven patients, including 4 patients of the unilateral VUSF and 3 patients of the bilateral VUSF, were diagnosed as sacral nerve injury with Gibbons grade II [15].

**Table 1:** Patients’ demographics and clinical data

Variables	*n* = 21	Range/percent
Age (years), M ± SD	40.5 ± 15.2	18–77
Gender, n		
Male	15	71.4%
Female	6	28.6%
Mechanism of injury, n		
Fall from height	15	71.4%
Traffic accident	4	19.0%
Crushing injury	2	9.5%
Tile classification, n		
Type C1	12	57.1%
Type C2	2	9.5%
Type C3	7	33.3%
Sacral fracture sides, n		
Left	6	28.6%
Right	8	38.1%
Bilateral	7	33.3%
Anterior pelvic ring injuries, n		
Single rami fracture	15	71.4%
Single symphyseal separation	4	19.0%
Rami fracture and symphyseal separation	1	4.8%
No	0	
Preoperative displacement of the sacral fracture (mm), M ± SD	19.7 ± 7.3	11–46

M, mean; SD, standard deviation

Fifteen males and six females were in the study, with the age at a mean of 40.5 ± 15.2 years (range 18–77 years). Patients underwent robot-aided percutaneous triangular osteosynthesis with an average of 9.0 ± 4.9 days (range 3–20 days) post-injury. The delay in pelvic surgery was due to the instability of the general condition and the prioritized treatment of concomitant injuries.

**Fig. 2:**
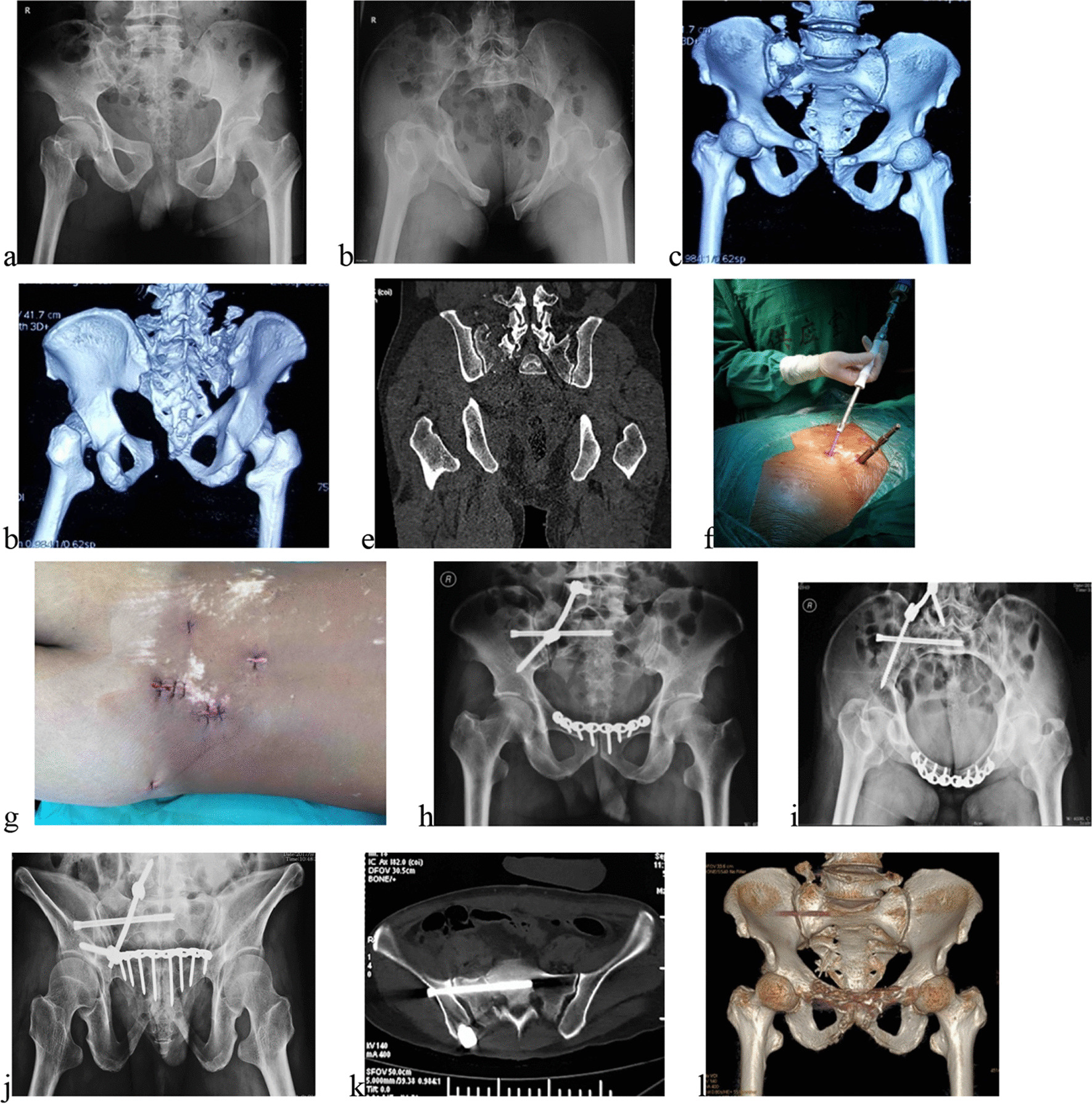
Male, 48 years old, a fall injury. **a**, **b** Anteroposterior and inlet pelvic views after admission. **c**, **d** 3D CT reconstruction: a Tile C1 pelvic ring injury. **e** The coronal view showing the right S1 facet fracture. **f** The percutaneous implantation of an iliac screw. **g** The sutured incisions. **h**–**j** Anteroposterior, inlet and outlet pelvic views after surgery. **k** The axial view revealing the good position of the iliosacral screw. **l** 3D CT reconstruction at one year demonstrating the union of the pelvic fracture

**Fig. 3:**
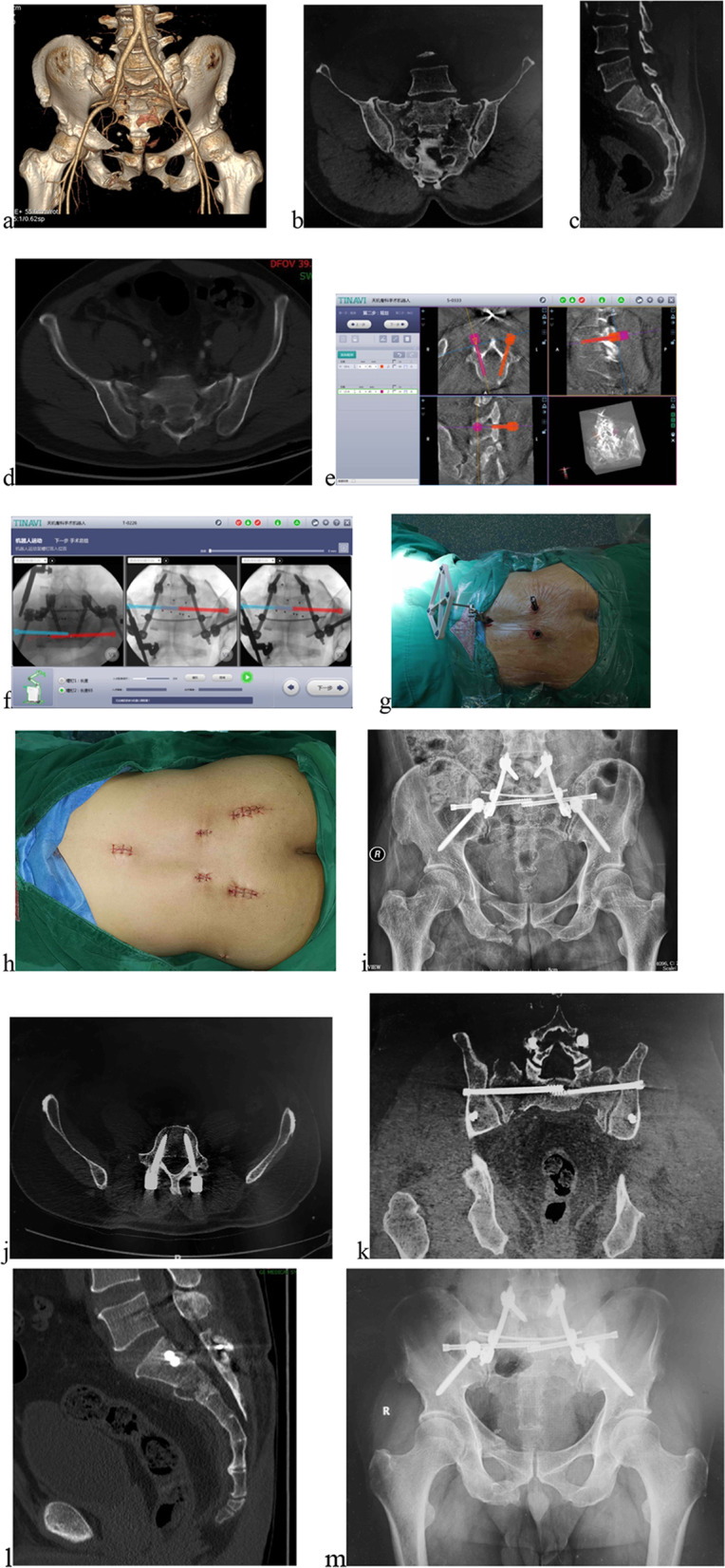
Male, 46 years old, a fall injury. **a**–**d** 3D CT reconstruction, coronal, sagittal and axial views after admission showing a type “H” sacral fracture associated with pubic superior and inferior rami fractures. **e**, **f** Planning of trajectories on bilateral L5 pedicle screws and bilateral S1 iliosacral screws. **g** Percutaneous pedicle screw implantation. **h** All sutured incisions. **i** Postoperative radiograph revealing bilateral triangular osteosynthesis for sacral fractures. **j**–**l** Postoperative CT views showing screws with good position. **m** Anteroposterior pelvic X-ray at one year after surgery demonstrating fracture healing

Of the 21 patients, 14 (66.7%) with the unilateral VUSF underwent unilateral triangular osteosynthesis fixation (Fig. 2), and the remaining 7 (33.3%) with the bilateral VUSF underwent bilateral triangular osteosynthesis fixation (Fig. 3). Operation time of posterior pelvic ring was a mean of 111.4 ± 31.5 min (range 70–180 min), and the intraoperative estimated blood loss was a mean of 110.5 ± 39.6 ml (range 60–210 ml) (Table 2). Operation time of anterior pelvic ring was a mean of 38.3 ± 16.6 min (range 15–70 min), and the intraoperative estimated blood loss was a mean of 85.0 ± 68.3 ml (range 10–250 ml). A total of 58 screws were inserted with robotic assistance in 21 patients, including 29 screws in L5 pedicles and 29 iliosacral screws in S1 vertebrae. In addition, there were 29 iliac screws that were inserted into the iliac channel between the inner and outer lamina with free hand technique. Among anterior pelvic ring injuries in 20 patients, 16 underwent surgical treatment while 4 underwent conservative treatment. Open reduction and plating fixation were performed through the Stoppa approach in 8 patients, close reduction and percutaneous screw fixation was performed in 6 patients, and anterior internal fixator (INFIX) fixation was utilized in the rest 2 patients.

**Table 2:** Perioperative data and postoperative results

Characteristics	n = 21	Range/percent
Duration from trauma to surgery (day), M ± SD	9.0 ± 4.9	3–20
Surgical time of posterior pelvic ring (min), M ± SD	111.4 ± 31.5	70–180
The intraoperative blood loss of posterior pelvic ring (ml), M ± SD	110.5 ± 39.6	60–210
Fluoroscopy frequency, M ± SD	24.8 ± 8.6	16–40
Fixation modes of anterior pelvic ring injury, n		
Plating fixation	8	38.1%
Screw fixation	6	28.6%
INFIX fixation	2	9.5%
Conservative treatment	4	19.0%
Postoperative displacement of the sacral fracture (mm), M ± SD	3.1 ± 2.7	0–10
Matta criterion, n		
Excellent	13	61.9%
Good	8	38.1%
Surgery-related complications, n		
Nonunion of the sacral fracture	1	4.8%
Hospital stay(day)	17.1 ± 5.5	9–32
Follow up (month)	18.1 ± 4.7	12–30
Healing time of sacral fractures (month)	4.8 ± 1.0	2–6
Majeed score, M ± SD	89.6 ± 4.4	80–97

M, mean; SD, standard deviation

The average maximal displacement of the sacral fracture was reduced from 19.7 ± 7.3 mm (range 11–46 mm) preoperatively to 3.1 ± 2.7 mm (range 0–10 mm) postoperatively. According to Matta’s criterion [16], the reduction quality was graded with “excellent” in 13 patients and “good” in 8 patients.

The length of the L5 pedicle screw, the iliac screw and the S1 iliosacral screw was 45 mm, 80–100 mm and 65–100 mm, respectively. The accuracy of robot-aided screw placement was verified by comparing the intraoperative planned position with the actual position, and the maximal angulation and displacement deviations were at a mean of 4.2° ± 2.5° and 1.7 ± 0.9 mm, respectively. Of those robot-aided inserted screws, in addition to 4 pedicle screws and 2 iliosacral screws cutting the cortical bone, the rest were all in the cancellous bone. The average fluoroscopy frequency of robot-aided screw insertion was 24.8 ± 8.6 (range 16–40), among which the fluoroscopy frequency of pedicle screws and iliac screws for ipsilateral sacral fractures was 19.1 ± 2.4, and that for bilateral sacral fractures was 36.1 ± 3.0. The success rate for one-time screw insertion with robotic assistance was 70.7% (41/58). The percentage of puncturing the iliac inner or outer lamina with free hand iliac screws was 10.3% (3/29), no neurovascular complications occurred.

No additional neurological impairment occurred intraoperatively. No wound infection, hardware failure and reduction loss occurred postoperatively. All patients were followed up for a minimum of 12 months, with a mean follow-up of 18.1 ± 4.7 months (range 12–30 months). After conservative treatment with oral drugs, sacral nerve impairment of 7 patients improved from Gibbons grade II to I at 3–12 months postoperatively. Combining radiology and clinic at the final follow-up, one patient was diagnosed as the nonunion of the sacral fracture, and the range of healing time in other patients was 2–6 months. Fifteen patients had their implants removed between one and two years after surgery, while we had offered all patients to undertake the implant removal. The other 6 patients did not wish to interfere with their implants, because they had not experienced significant discomfort in the surgical area or due to their advanced ages. Based on Majeed scoring system of functional evaluation [17], the clinical outcome at the final follow-up were “excellent” in 18 patients and “good” in 3 patients, with the mean score of 89.6 ± 4.4 points (range 80–97).

## Discussion

Triangular osteosynthesis allows for percutaneous application, which has been attempted initially in experienced hands [18]. However, minimally invasive treatment for posterior pelvic ring injuries with vertical displacement is challenging due to the intractability of free hand screw implantation and the close reduction technique [19]. The robot navigation system takes advantage of the combination of navigation planning and real-time positioning, assisting accurate percutaneous screw placement. In addition, minimally invasive pelvic surgery with robotic assistance precludes the need for extensive soft tissue dissection, offering decreasing of intraoperative blood loss and wound-related complications [10]. Until now, the robot-aided triangular osteosynthesis for patients with VUSFs has not, to our knowledge, been described in literature. Based on our findings, we report that robot-aided percutaneous triangular osteosynthesis technique is safe and reliable for the treatment of VUSFs.

In order to regain pelvic construct and avoid implant failure, anatomical and radiological principles are indispensable. With regards to the vertically displaced sacral fracture, reasonable utilization of reduction instruments and the considerable countertraction required intraoperatively are critical for closed reduction, even with robotic assistance. A recent study demonstrated that the better reduction of the posterior pelvic fractures the higher Majeed score (*P* = 0.013) [20]. Different from the conventional open procedures, all the patients of VUSFs in this study underwent minimally invasive surgeries with TiRobot assistance on posterior pelvic rings. Therefore, the reduction process is highly essential. First of all, the vertical displacement of the posterior pelvic ring needs to be effectively corrected by the longitudinal countertraction associated with the application of the distraction clamp, because a fundamental requirement for iliosacral screw fixation is to obtain an anatomical reduction of the screw corridor. Compared with the conventional open operation for VUSFs, closed reduction, keeping the least possible invasiveness, still has some challenges even with fluoroscopy by using the C-arm machine. The inadequate reduction must be deliberately considered when the robot-aided percutaneous technique was applied, particularly in the delayed VUSFs. If the placement of the rod connection was overly oblique due to the position of the pedicle screw and the iliac screw, an offset short connector between the rod and the iliac screw should be applied for better vertical reduction. Even if the offset short connector was installed, the operation time was not significantly prolonged based on our experience. Secondly, reduction of the rotation deformity and the transverse separation can be corrected by using the Schanz pin as a joystick combined with a ball spike pusher acting on the iliac outer lamina. Thirdly, bilateral trajectories of L5 pedicle screws and S1 iliosacral screws can be planned simultaneously to save the operation time. According to our case records, the operation time of bilateral fixation was less than double of that in the unilateral injuries, although the operation procedure of the former is twice than that of the latter. Meanwhile, in light of the intraoperative blood loss and operation time in the previous reports with the free hand technique, the comparative results in this study declined significantly, not only in similar patients with the unilateral VUSF but also the bilateral VUSF [18, 21, 22], which demonstrated advantages of the robot-aided technique.

We advocate robot-aided screw implantation primarily in the fixation of posterior pelvic ring injuries, because if there is a potential risk of screw penetration during the guide wire insertion, the caution on the trajectory can be achieved automatically in real time while drilling. Consequently, robot-aided screw insertion has an extremely low incidence of cutting (6/58, 10.3%) or penetrating (0/58, 0%) the cortical bone, and the success rate for one-time screw insertion with robotic guidance was 70.7% (41/58). Fluoroscopic conditions due to the individualized projecting angulation and intestinal gas accumulation must be considered for higher success rate. Another reason for the success rate less than 100% is that we have too high demands for the position of screw placement to avoid the disastrous consequences such as neurovascular injuries, rather than the problem of the TiRobot system. Nevertheless, based on our previous attempts and the existing study [23], robot-aided iliac screw insertion in the iliac wing didn’t show significant advantages compared to that with free hand technique. Conversely, iliac screw implantation with robotic assistance did enhance the operation time and the fluoroscopy frequency. Of course, whether open or percutaneous procedure was performed, part of the entry-point at PSIS must be removed to form a small bone depression, so as to avoid postoperative skin irritation caused by implant prominence. In light of the application of the S2-alar-iliac (S2AI) screw in the treatment of scoliosis, this risk can be significantly reduced [24]. Furthermore, unlike the placement of the iliac screw, the S2AI screw doesn't necessitate the assistance of offset connectors, which may further shorten the operation time.

Based upon our observation in the cases, fracture gaps existed in the majority of VUSF preoperatively. However, sacral fracture nonunion (8%) is commonly felt to be technical problems arising from excessive dissection unsatisfactory reduction [1]. If spinopelvic fixation connectors were locked in the process of iliosacral screw insertion, it resisted compression between the sacral fracture ends. As one component of triangular osteosynthesis, the partially threaded screw necessitates to eliminate the horizontal gap in sacral fractures, which is the main reason for only one case of sacral fracture nonunion in this study. However, attention should be paid to sacral fractures involving foramina to avoid nerve entrapment caused by over-compression. No sacral laminectomy or decompression was performed in all patients due to the limitation of inclusion criterion. For patients with Gibbons grade III or IV sacral nerve injury who meet the clear indication of nerve exploration, open reduction is routinely performed instead of percutaneous surgery. In this study, the neurological function was significantly improved in 7 patients with incomplete nerve damage after robot-aided triangular osteosynthesis, indirectly indicating that good reduction and stabilization of VUSFs was conducive to the nerve recovery by reconstructing the nerve corridors.

Moreover, in addition to having stronger mechanical strength than single iliosacral screw fixation [3], triangular osteosynthesis has two special advantages. One has the dual function of simultaneous reduction and fixation, and the other is to treat such injuries involving lumbosacral junctions, such as L5/S1 facet joints. Therefore, percutaneous triangular osteosynthesis is suitable for all VUSFs, except for those undisplaced cases with intact lumbosacral junctions. Whereas, a compromise between satisfactory reduction and mechanically stable fixation has to be considered for VUSF patients with low bone mineral density. As shown in a previous study of our team [11], injecting the bone substitute into screw corridors has the advantage of enhancing the fixation strength in patients with osteoporosis without increasing additional invasiveness. Under the guidance of TiRobot system in this study, one patient with the pelvic fragility fracture underwent bone grafting in the S1 corridor followed by iliosacral screw fixation, achieving bone healing and satisfactory function, although the reduction quality was good according to Matta’s criterion.

Certain limitations need to be taken into account on the results of this study. Our analysis relies on this TiRobot system, and it is necessary to verify whether similar findings would be obtained by using different robot devices. The orthopedic robot may only be generalizable to large medical institution with high patient flow due to the device cost that is the main barrier. Also, the lack of comparison between the robot-aided percutaneous technique and the conventional open method may decrease persuasion. The accumulation of cases is warranted to provide additional evidence, and further research can benefit from multicenter cooperation to accurately assess the results of patients for guiding clinical applications.

## Conclusions

The increased attention given to minimally invasive surgery in pelvic fractures is justified by the great invasiveness of conventional open approaches, the development of the imaging equipment and the improvement of the surgical technique. Our study reveals that percutaneous triangular osteosynthesis with robotic assistance is safe and reliable for patients with the fresh VUSF, which produces an intraoperative screw trajectory comparable to the position achieved with the postoperative CT scans. In addition, the technique may help to reduce incision-related complications in the treatment of such complex sacrum fractures.

## Data Availability

The datasets used or analyzed during the current study are available from the corresponding author on reasonable request.
